# Efficacy, Safety, and Pharmacokinetics of a Novel Human Immune Globulin Subcutaneous, 20 % in Patients with Primary Immunodeficiency Diseases in North America

**DOI:** 10.1007/s10875-016-0327-9

**Published:** 2016-08-31

**Authors:** Daniel Suez, Mark Stein, Sudhir Gupta, Iftikhar Hussain, Isaac Melamed, Kenneth Paris, Amy Darter, Christelle Bourgeois, Sandor Fritsch, Heinz Leibl, Barbara McCoy, David Gelmont, Leman Yel

**Affiliations:** 1 Allergy, Asthma and Immunology Clinic PA, Irving, TX USA; 2 Allergy Associates of the Palm Beaches, North Palm Beach, FL USA; 3 Division of Basic and Clinical Immunology, University of California at Irvine, Irvine, CA USA; 4 Vital Prospects Clinical Research Institute, Tulsa, OK USA; 5 IMMUNOe International Research Centers, Centennial, CO USA; 6 LSU Health Sciences Center, Children’s Hospital, New Orleans, LA USA; 7 Oklahoma Institute of Allergy and Asthma Clinical Research, LLC, Oklahoma City, OK USA; 8 Baxalta Innovations GmbH, now part of Shire, Vienna, Austria; 9 Baxalta US Inc., now part of Shire, 650 East Kendall Street, Cambridge, MA USA; 10 Baxalta US Inc., now part of Shire, Westlake Village, CA USA

**Keywords:** Primary immunodeficiency diseases, Immunoglobulin replacement therapy, Subcutaneous administration, 20 % immunoglobulin, Pharmacokinetics

## Abstract

Patients with primary immunodeficiency disease (PIDD) typically require life-long intravenous (IV) or subcutaneous (SC) immunoglobulin (Ig) replacement therapy to prevent recurrent infections. The efficacy, safety, and pharmacokinetics of a highly concentrated (20 %) Ig preparation for SC administration (IGSC 20 %) were evaluated in a prospective trial in patients with PIDD. A total of 74 patients (aged 3–83 years) received 4327 IGSC 20 % infusions over a median of 380.5 days. The rate of validated serious bacterial infections was 0.012 event/patient-year (*p* < 0.0001 compared with the historical control), and the annualized rate of infection was 2.41 events/patient. Median IgG trough levels were >14.5 g/l. The median maximum infusion rate was 60 ml/h/site (range 4.4–180), resulting in a median infusion duration of 0.95 h. A volume ≥30 ml was infused per site in 74.8 % of IGSC 20 % infusions. Most (84.9 %) infusions were administered using ≤2 infusion sites; for 99.8 % of infusions, there was no need to interrupt/stop administration or reduce the infusion rate. No related serious adverse event (AE) occurred during IGSC 20 % treatment; related non-serious AEs occurred at a rate of 0.036 event/infusion. The incidence of related local AEs was 0.015 event/infusion and of related systemic AEs was 0.021 event/infusion; most were mild in severity, none severe. Increased infusion rates or volumes were not associated with higher AE rates. The investigated IGSC 20 % treatment was shown to be effective and safe, enabling higher infusion rates and volumes per site compared to conventional SC treatments, resulting in fewer infusion sites and shorter infusion durations.

**Electronic supplementary material:**

The online version of this article (doi:10.1007/s10875-016-0327-9) contains supplementary material, which is available to authorized users.

## Introduction

Primary immunodeficiency diseases (PIDD) result from genetic defects in the immune system, more than 300 of which have been identified [1]. Patients with PIDD are susceptible to bacterial, viral, and fungal infections [2]. In about 53 % of these patients, the diagnosis of PIDD is associated with defective antibody production with or without decreased levels of serum immunoglobulin (Ig) (e.g., common variable immunodeficiency, specific antibody deficiency, X-linked or autosomal recessive agammaglobulinemia, hyper IgM syndrome) [3].

Polyclonal IgG preparations purified from human plasma have been used as antibody replacement therapy to reduce the number and severity of infections in patients with PIDD since the early 1950s [4, 5]. Due to their diverse specificity, polyclonal IgGs are able to neutralize infectious agents, enhance phagocytosis, and modulate the immune response. For clinical effectiveness in PIDD, antibody replacement therapy generally requires a monthly dose in the range of 0.3 to 0.6 g/kg body weight (BW) administered intravenously (IV) or subcutaneously (SC) [6]. With IgG solutions at 5 or 10 % weight per volume (*w*/*v*) protein concentration, volumes of 3 to 6 ml/kg BW are routinely administered IV [7].

IgG replacement therapy administered SC (IGSC) has been widely established in recent years. It is safe and well-tolerated with a particularly low risk of systemic adverse reactions compared to IV administration of IgG [5, 7]. IGSC slowly diffuses from the SC space into the systemic circulation while equilibrating with the extravascular compartment. Consequently, there is no high peak in the IgG concentration as seen following IV infusion, and sustained steady-state IgG levels can be achieved. At the same monthly equivalent dose as IV immunoglobulin replacement therapy (IGIV), IGSC may lead to higher serum IgG trough levels compared to IV infusion [8, 9]. With appropriate training by healthcare professionals, SC infusions of immunoglobulin can easily be performed by patients at home without assistance, thus increasing their comfort and independence and also reducing cost [10].

A drawback of IGSC compared to IV infusion is the limited volume that can be administered into each site due to resistance of the SC extracellular matrix. Consequently, the rate at which IGSC can be infused is slower compared to IGIV, resulting in longer infusion times and requiring multiple infusion sites per treatment, usually on a weekly basis. One of the strategies to overcome these limitations is the development of highly concentrated IgG formulations that allow for infusion of the same dose in smaller infusion volumes compared to less concentrated products [5].

Alternatively, SC immunoglobulin product that could be infused at higher rates and volumes per site would provide an advantage over currently available conventional SC preparations by decreasing infusion duration and the number of infusion sites. Immune globulin subcutaneous (human) (IGSC 20 %, Baxalta, now part of Shire) is a ready-for-use, sterile liquid preparation of highly purified, concentrated, functionally intact human IgG developed specifically for SC administration to provide patients with an additional treatment option.

Presented here are the results of a prospective phase 2/3 study that evaluated the efficacy, safety, tolerability, and pharmacokinetic (PK) characteristics of a new IGSC 20 % treatment option in adult and pediatric patients with PIDD in North America.

## Methods

### Study Design

This prospective, open-label clinical trial (registered on clinicaltrials.gov: NCT no. 01218438) was conducted in accordance with the Declaration of Helsinki and the international standards of Good Clinical Practice. Patients were enrolled at 15 sites in the USA and Canada; informed consent was obtained from each patient prior to undergoing any study procedures.

The trial comprised four study periods: in period 1, patients received IGIV 10 % and in periods 2 to 4, they received IGSC 20 % (Supplementary material Figure S1). Period 1 was designed mainly to determine the area under the IgG concentration curve (AUC) following IV administration (AUC_IV_). During IGSC 20 % treatment, systemic exposure equivalent to previous IGIV 10 % treatment (as measured by the AUC of total IgG over time) was targeted. Therefore, IGSC 20 % doses were adjusted to compensate for the lower bioavailability of IgG-administered SC. The adjustment factor to calculate the IGSC 20 % dose in period 2 (145 %) was approximated from the PK data of available IGSC products [11, 12]. Next, based on the PK data collected from the first 18 patients treated with IGSC 20 % at 145 % of the IGIV 10 % dose in period 2, the IGSC 20 % dose that would (on average) provide equivalent IgG exposure as IGIV 10 % administration (“adjusted IGSC 20 % dose”) was determined to be 145 % of the IGIV 10 % dose. In period 3, patients were treated with IGSC 20 % at the “adjusted dose.” Since this adjusted dose represented the average dose-response of only 18 patients, the possibility of over- or under-dosing could not be excluded. Thus, for each patient, an individually adapted (individualized) dose of IGSC 20 % was determined by comparing the patient trough level (equivalent to the steady-state serum IgG level) attained in period 3 to the expected trough level increase calculated from the PK data of periods 1 and 2. During period 4, the patients were infused with IGSC 20 % at this dose for 40 weeks.

### Study Population

Patients aged 2 years and older diagnosed with PIDD involving defective antibody production and requiring IgG replacement as defined by the International Union of Immunological Societies (IUIS) Scientific Committee 2011 [13] and by diagnostic criteria according to Conley et al. [14] were eligible for enrollment in the study. Inclusion criteria also required that patients had received a stable dose (IV or SC) of IgG equivalent to at least 0.3 g/kg BW/4 weeks and not higher than 1 g/kg BW/4 weeks for a minimum of 12 weeks prior to first treatment and had serum IgG trough levels >5 g/l at screening. Patients were excluded from the study if they had a history of hepatitis B or C or a positive human immunodeficiency virus test; if they had persistent abnormal alanine aminotransferase and aspartate aminotransferase values >2.5 times the upper limit of normal for the testing laboratory, creatinine clearance value <60 % of normal according to their age and gender, or severe neutropenia or protein loss at screening; or if they had been diagnosed with a malignancy, were receiving anticoagulation therapy, or had a history of thrombotic episodes. Patients were also excluded if they were receiving antibiotics, had an active infection at the time of screening, or had had an acute serious bacterial infection within 3 months prior to screening. A complete list of eligibility criteria is available in the Supplementary material.

### Study Product

IGSC 20 % is a liquid concentrate of functionally intact, aggregate-free IgG derived from human plasma. The production of IGSC 20 % follows the same manufacturing processes as IGI, 10 % solution (marketed under the Baxalta, now part of Shire trade-name GAMMAGARD LIQUID® in the US and Kiovig® in the EU) except for ultra/diafiltration and final formulation at 20 % (*w*/*v*) protein concentration. The manufacturing process of IGSC 20 % includes three dedicated virus inactivation and reduction steps: solvent/detergent (S/D) treatment [15], nanofiltration (35 nm) [15, 16], and low pH incubation with elevated temperature [17, 18]. Similar to IGI, 10 %, IGSC 20 % contains glycine as stabilizer to minimize IgG dimerization. The final IGSC 20 % product has a viscosity of 14.4 mPa/s, an osmolality of 280–292 mOsm/kg and contains trace amounts of IgA (average concentration 80 μg/ml). Each lot of IGSC 20 % is monitored for procoagulant activity using a thrombin generation assay to ensure that the final container is free of procoagulants.

### Immunoglobulin Treatments

Patients received IGIV 10 % at the monthly equivalent dose used prior to entering the study (required dose range 0.3-1.0 g/kg BW/4 weeks) every 3 or 4 weeks at the clinical site. IGSC 20 % was administered once a week; doses used in the respective study periods are described in the “Study design” section. IGSC 20 % was infused using an electromechanical syringe-driver pump (CME T34L, Caesarea Medical Electronics) and high-flow, 24-gauge low-resistance needles (RMS Medical Products). The needle sets used in the trial ranged from 6 to 12 mm in length at the discretion of the investigator; there was no specified needle length for infusion. Infusion rates were increased incrementally: the initial two infusions were to be started at 10 ml/h/infusion site and could be increased to a maximum of 20 ml/h/infusion site. Subsequent infusions could begin at the maximum tolerated infusion rate for the initial infusions, and as tolerated, the infusion rate was to be increased to a maximum of 60 ml/h/site. For patients with a body weight of 40 kg or above, an infusion volume of up to 60 ml was to be administered per infusion site if well tolerated. For patients with a body weight below 40 kg, it was recommended to limit infusion volume to 20 ml per site for the initial two infusions. Volumes could then be increased to a maximum of 60 ml per site as tolerated. Multiple infusion sites could be used simultaneously. Infusion sites were to be rotated to avoid any single infusion site being used repeatedly within a short time interval. Infusion of IGSC 20 % at home was possible after sufficient training of the patient/caregiver or with assistance of a healthcare professional.

### Efficacy Assessment

Efficacy was evaluated based on the analysis of serious acute bacterial infections, all infections, and IgG levels. Serious bacterial infections, such as bacteremia/sepsis, bacterial meningitis, osteomyelitis/septic arthritis, bacterial pneumonia, and visceral abscesses caused by a recognized bacterial pathogen were diagnosed according to the Diagnostic Criteria for Serious Infection Types in the FDA Guidance for Industry, June 2008 [19]. The primary efficacy assessment was the annualized rate of validated acute serious bacterial infections (VASBIs, defined as R_VASBI_ = mean number of VASBI/patient/year). Efficacy was also evaluated by the annualized rate of all infections (i.e., VASBIs and all other events clinically assessed as infections), as well as the number of fever episodes (body temperature ≥ 38 °C), the number of days with fever, the number of days missed from school/work or to perform normal daily activities due to illness/infection, the number of admissions to a hospital as an in-patient, and the number of days as a stationary patient in the hospital, as well as urgent or unscheduled physician visits due to illness/infection (apart from the regular investigator/study site visits scheduled every 8–12 weeks within the study).

### Safety

Safety was evaluated through clinical and laboratory assessments. Safety data were collected throughout the study. The AEs that occurred during the infusions at the site (every 8–12 weeks) were recorded by the investigator. All investigators were specifically trained on symptoms of potential AEs. All patients received an eDiary tablet to continuously record home treatments, AEs, and additional information as they occurred. The investigator provided guidance for the patient/caregiver regarding identification and documentation of local and systemic AEs, including signs of hemolysis such as fever, chills, back pain, fatigue, and dark urine. All patients were instructed to inform the investigator/site immediately in case of such an event. In addition, the patient was contacted by the investigator within 3–5 days after each infusion, either at the study site or at their home for follow-up to ensure appropriate documentation of AEs. The investigators reviewed patients’ eDiary entries at every site visit. All AEs were assessed by the investigator using comprehensive data collection systems—including the patient’s eDiary—for seriousness, severity, temporal association, and possible causal relatedness to the investigational product.

Monitoring for potential cases of hemolysis comprised routine hematology screening and hemolysis screening as recommended by the FDA Guidance for Industry (June 2008 [19]). If a decrease of hemoglobin ≥2 g/dl was measured during either the hematology or hemolysis screening, the assessments to monitor for potential cases of hemolysis were to be performed within 48–72 h of being informed of the hemoglobin level, unless there was a clear alternative explanation. These assessments included direct antiglobin (Coomb’s) test, plasma-free hemoglobin, reticulocyte count, lactate dehydrogenase (LDH), serum haptoglobin, and urine hemosiderin.

### Pharmacokinetics

Determination of total serum IgG concentration was performed at a central laboratory using a validated enzyme-linked immunosorbent assay (ELISA)-based method. PK assessments were performed over a specific dosing interval: in period 1 between infusions 10 and 11, for patients aged 12 years and older; in period 2 between infusions 9 and 10, for the first 18 patients aged 12 years and older; and for all patients in period 4 between infusions 17 and 18 (Supplementary material Figure S1). In period 2 and 4, samples were collected preinfusion and on days 1, 3, 5, and 7 after IGSC 20 % administration in patients aged 12 years and older. In patients aged 2–11 years, samples to determine the AUC over a dosing interval (AUC_0−τ_) after IGSC 20 % administration were only drawn preinfusion and on days 3 and 7 in order to limit the number of blood draws.

Total serum IgG trough levels were assessed immediately prior to each IGIV 10 % infusion in period 1 and during IGSC 20 % treatment: prior to infusion 1, twice each in periods 2 and 3, four times during period 4, and at the end-of-study visit.

### Statistical Methods

Efficacy was assessed by the mean number of VASBIs per patient per year (R_VASBI_)_._ Assuming R_VASBI_ = 0.6 and a one-sided test and a Type I error = 0.01, a sample size of 59 patients would have in excess of 85 % power to test the null hypothesis that R_VASBI_ ≥1.0 against the alternative hypothesis R_VASBI_ <1.0. R_VASBI_ and 99 % upper confidence limit (CI) were calculated using a Poisson model accounting for the length of the observation periods per patient.

The AUC between adjacent infusions was calculated by the trapezoidal rule. To allow for comparisons between periods 1, 2, and 4, AUC_0−_τ was standardized for the infusion intervals (3 or 4 weeks vs. 1 week = AUC_0−τ;h_;). The bioavailability of IGSC 20 % relative to IGIV 10 % was estimated from the ratio of AUC_0−τ;h_ in period 4 (SC treatment at an individualized dose, once per week) over AUC_0−τ;h_ in period 1 (IV treatment every 3 or 4 weeks) standardized to 1 week.

### Measures of Patient Experience

Treatment burden related to Ig therapy was evaluated with the Life Quality Index questionnaire (LQI) for the age group 2 to 12 years (observer: parent) and the age group 13 years and older (observer: patient) [20, 21]. The LQI covers four domains: treatment interference, therapy-related problems, therapy settings, and treatment costs. While data related to treatment cost LQI domain were collected, they were not included in the analysis as patients received free treatment during the study. Treatment satisfaction was surveyed in age groups from 2 to 12 years (observer: parent) and 13 years and older (observer: patient) using the Treatment Satisfaction Questionnaire for Medication (TSQM-9) [22]. Evaluations were performed at baseline, at the end of periods 1 and 3 and during the end-of-study visit (or early termination visit). Score changes between end of period 1 and end of period 3 or end of study visit were analyzed. In both the LQI and TSQM-9, higher scores indicated higher satisfaction.

## Results

### Study Population

Seventy-seven (77) patients with PIDD started study period 1 (51.9 % males, 48.1 % females; age range 3–83 years; Supplementary material Table S1). More than half of the patients had either common variable immunodeficiency (33.7 %), “specific antibody deficiency” (23.4 %), or agammaglobulinemia (14.3 %, congenital and autosomal recessive combined; Supplementary material Table S2). All patients had received antibody replacement therapy until just prior to study entry (68.8 % IV; 31.2 % SC). None of the patients screened for inclusion in the study had elevated transaminases, and therefore, no patient was excluded based on these criteria. Overall, 74 patients were administered IGSC 20 % and 67 (90.5 %) patients completed the study (Supplementary material Figure S2). Two patients were discontinued from the study during period 1 for non-adherence, and one patient withdrew from the study after being hospitalized for mild headache (SAE) assessed as related to IGIV 10 % infusion. During IGSC 20 % treatment, seven patients prematurely discontinued treatment. One patient who had common variable immune deficiency and fibromyalgia experienced fatigue (unrelated to IGSC 20 % administration) and chose to discontinue. One patient was terminated from the study because of poor adherence to the study protocol and five patients withdrew consent for reasons unrelated to AEs (Supplementary material Figure S2).

### Efficacy

During IGSC 20 % treatment, the point estimate of R_VASBI_ was statistically significantly lower than 1 (R_VASBI_ = 0.01; 99 % CI = 0.024; *p* < 0.0001; Table 1). One VASBI of pneumonia treated with systemic antibiotics developed during period 4 in a 78-year-old patient who had specific antibody deficiency and a history of allergic bronchopulmonary aspergillosis.

**Table 1:** Efficacy of protection against infections

Parameter	Annualized rate^a^ per patient per treatment			
	IGIV 10 % (19.67 PY)^b^		IGSC 20 % (83.70 PY)^b^	
	Point estimate	95 % CI	Point estimate	95 % CI
Validated acute bacterial infections (VASBIs) [upper limit 99 % CI]	0.00 [0.234]	0.00 to 0.19	0.01^c^ [0.024]	0.01 to 0.02
All infections ^d^	3.86	2.77–5.22	2.41	1.89–3.03
Sinus infections	0.97	0.61–1.45	0.69	0.50–0.93
Fever episodes	0.61	0.34–0.99	0.13	0.08–0.21
Days off school or work due to illness or infection	3.20	1.88–5.03	1.16	0.70–1.79
Days on antibiotic**s**	63.2	43.39–88.29	57.59	40.71–78.59
Days in hospital	0.20	0.08–0.42	0.11	0.05–0.20
Hospitalizations	0.05	0.02–0.10	0.02	0.01–0.04
Acute physician /emergency room visits	1.73	1.03–2.68	0.86	0.54–1.28

*NA* not applicable, *n* number of treated patients, *CI* confidence interval

^a^Rate = number of infections divided by the total number of patient-years under treatment

^b^PY: Patient-years = number of patient-years under treatment

^c^For the null hypothesis of one or more validated ASBI per year, *p* value < 0.0001

^d^VASBIs and all other events clinically assessed as infections during the study

The point estimate of the annualized rate of all infections was 2.41 events/patient during IGSC 20 % treatment and 3.86 events/patient during IGIV 10 % administration (Table 1). While receiving IGSC 20 %, the annualized rate of days off of school/work was 1.16 days, and hospitalizations occurred at a rate of less than once per year for ≤1 day/year (all point estimates). Overall, 58/74 (78.4 %) patients received antibiotics mostly for treatment of acute infections during IGSC 20 % for an annualized duration of 57.59 days (point estimate). The point estimate of the rate of acute (urgent or unscheduled) physician visits due to infection or other illness was also less than one visit per year (Table 1).

### Safety

IGSC 20 % was safe, with no serious causal-related AEs. Of the three serious AEs that occurred during the trial, one was a mild headache assessed as related to IGIV 10 % by the investigator for which the patient was hospitalized and kept under observation and subsequently withdrew from the study. The other two SAEs, a severe lung adenocarcinoma and a moderate pneumonia, the VASBI described above, were not deemed related to IGSC 20 % treatment.

The incidence of non-serious AEs per infusion was 0.108 event/infusion during IGSC 20 % treatment and was 0.556 event/infusion during IGIV 10 % administration (Table 2). Of the 466 non-serious AEs (other than infections) reported for IGSC 20 %, 157 non-serious AEs (0.036 event/infusion) were deemed causally related to IGSC 20 %; most (136/157; 86.6 %) were of mild severity; none were severe.

**Table 2:** Summary of AE analyses

AE categories	Treatments			
	IGIV 10 %		IGSC 20 %	
	Number (%) of patients (*n* = 77)	Number (rate)^a^ of AEs (*n* = 324)	Number (%) of patients (*n* = 74)	Number (rate)^a^ of AEs (*n* = 4327)
Non-serious AEs (excluding infections)	51 (66.2)	180 (0.556)	57 (77.0)	466 (0.108)
Mild	43 (55.8)	141 (0.435)	53 (71.6)	360 (0.083)
Moderate	17 (22.1)	37 (0.114)	31 (41.9)	104 (0.024)
Severe	2 (2.6)	2 (0.006)	2 (2.7)	2 (<0.001)
Causally related non-serious AEs	28 (36.4)	80 (0.247)	28 (37.8)	157 (0.036)
Mild	22 (28.6)	59 (0.182)	24 (32.4)	136 (0.031)
Moderate	9 (11.7)	19 (0.059)	9 (12.2)	21 (0.005)
Severe	2 (2.6)	2 (0.006)	0 (NA)	0 (NA)
Causally related local non-serious AEs (excluding infections)	2 (2.6)	2 (0.006)	18 (24.3)	67 (0.015)
Mild	1 (1.3)	1 (0.003)	16 (21.6)	62 (0.014)
Moderate	1 (1.3)	1 (0.003)	3 (4.1)	5 (0.001)
Severe	0 (NA)	0 (NA)	0 (NA)	0 (NA)
Causally related systemic non-serious AEs (excluding infections)	27 (35.1)	78 (0.241)	19 (25.7)	90 (0.021)
Mild	22 (28.6)	58 (0.179)	16 (21.6)	74 (0.017)
Moderate	8 (10.4)	18 (0.056)	8 (10.8)	16 (0.004)
Severe	2 (2.6)	2 (0.006)	0 (NA)	0 (NA)
SAEs (including infections)	1 (1.3)	1 (0.003)	2 (2.7)	2 (<0.001)
Mild	1 (1.3)	1 (0.003)	0 (NA)	0 (NA)
Moderate	0 (NA)	0 (NA)	1 (1.35)	1 (<0.001)
Severe	0 (NA)	0 (NA)	1 (1.35)	1 (<0.001)
Causally related SAEs	1 (1.3)	1 (0.003)	0 (NA)	0 (NA)
Causally related AEs leading to discontinuation	1 (1.3)	1 (0.003)	0 (NA)	0 (NA)
AEs leading to death	0 (NA)	0 (NA)	0 (NA)	0 (NA)

*n* total number of patients or total number of infusions, *AE* adverse event, *NA* not applicable; *SAE* serious AE

^a^Rate per infusion = total number of AEs divided by the total number of infusions

Systemic AEs assessed as causally related to IGSC 20 % treatment were reported in 25.7 % of patients with an incidence of 0.021 event/infusion. The most frequent systemic AEs considered related to IGSC 20 % infusions were headache (0.011 event/infusion) followed by fatigue and nausea (0.002 event/infusion each; Table 3). Headache was experienced by 10.8 % of patients receiving IGSC 20 % infusion. Diarrhea was reported by 2.7 % of patients, however with an incidence of less than 0.001 per infusion. The other systemic AEs deemed related to IGSC 20 % were reported at a very low frequency (≤0.001 event/ infusion, Table 3). There was no event of laboratory-confirmed hemolysis following IGSC 20 % administration. A decline in hemoglobin of 2.0 g/dl or more was observed in six patients (during IGIV 10 % treatment (*n* = 1), during IGSC 20 % administration (*n* = 3), and at the “end-of-study” visit (*n* = 2)). However, at no time was there a concordance of other laboratory test results (e.g., Coomb’s test, haptoglobin, free hemoglobin, LDH, urine hemosiderin) supporting a diagnosis of hemolysis in these patients. No renal AEs or changes in laboratory values that measure kidney function were reported during the study.

**Table 3:** Causally related non-serious AEs during the IGSC 20 % treatment

Adverse event^a^	% of patients^c^ *N* = 74	Rate per infusion^d^ *N* = 4327
Causally related^b^ systemic AEs	25.7	0.021
Headache	10.8	0.011
Fatigue	6.8	0.002
Nausea	6.8	0.002
Diarrhea	2.7	<0.001
Myalgia	4.1	0.001
Dizziness	2.7	0.001
Migraine	2.7	<0.001
Somnolence	2.7	<0.001
Abdominal pain lower	1.4	<0.001
Anti-GAD antibody positive	1.4	<0.001
Pain	1.4	<0.001
Pruritus	1.4	<0.001
Causally related^b^ local AEs	24.3	0.016
Infusion site erythema (including Injection site erythema)	10.8	0.005
Infusion site pain (including Infusion site discomfort and Injection site pain)	16.2	0.008
Infusion site pruritus (including Injection site pruritus)	4.1	<0.001
Infusion site urticaria	2.7	<0.001
Burning sensation	1.4	<0.001
Infusion site edema	1.4	<0.001
Urticaria	1.4	<0.001

^a^AEs excluding infections

^b^Related AE as assessed by the investigator. Missing relationships were treated as related

^**c**^% of patients = (total number of affected patients divided by the total number of patients under treatment) × 100

^d^Rate per infusion = total number of AEs divided by the total number of infusions under treatment

Causally related local AEs occurred at a frequency of 0.015 event/infusion during IGSC 20 % treatment (Table 2); only 58/4327 (1.3 %) IGSC 20 % infusions were associated with one or more related local AEs. Over the entire course of the study, 24.3 % of patients reported experiencing one or more local AEs related to IGSC 20 % infusion at some time during the study (Table 2). However, the proportion of patients reporting one or more related local AE and the incidence of related local AEs per patient per year decreased throughout the study. At infusion 1, 13 % of the patients reported one or more related local AE; by infusion 6, this proportion dropped to below 5 % of the patients and continued to decrease to around 2 % or below and remained at this level until the end of the study (Supplementary material Figure S3A). In addition, the annualized rate of related local AEs was highest in the first 4 weeks of IGSC 20 % treatment and decreased progressively thereafter to values below 1 after 16 weeks of treatment (Supplementary material Figure S3B).

### IGSC 20 % Administration Characteristics

**Fig. 1:**
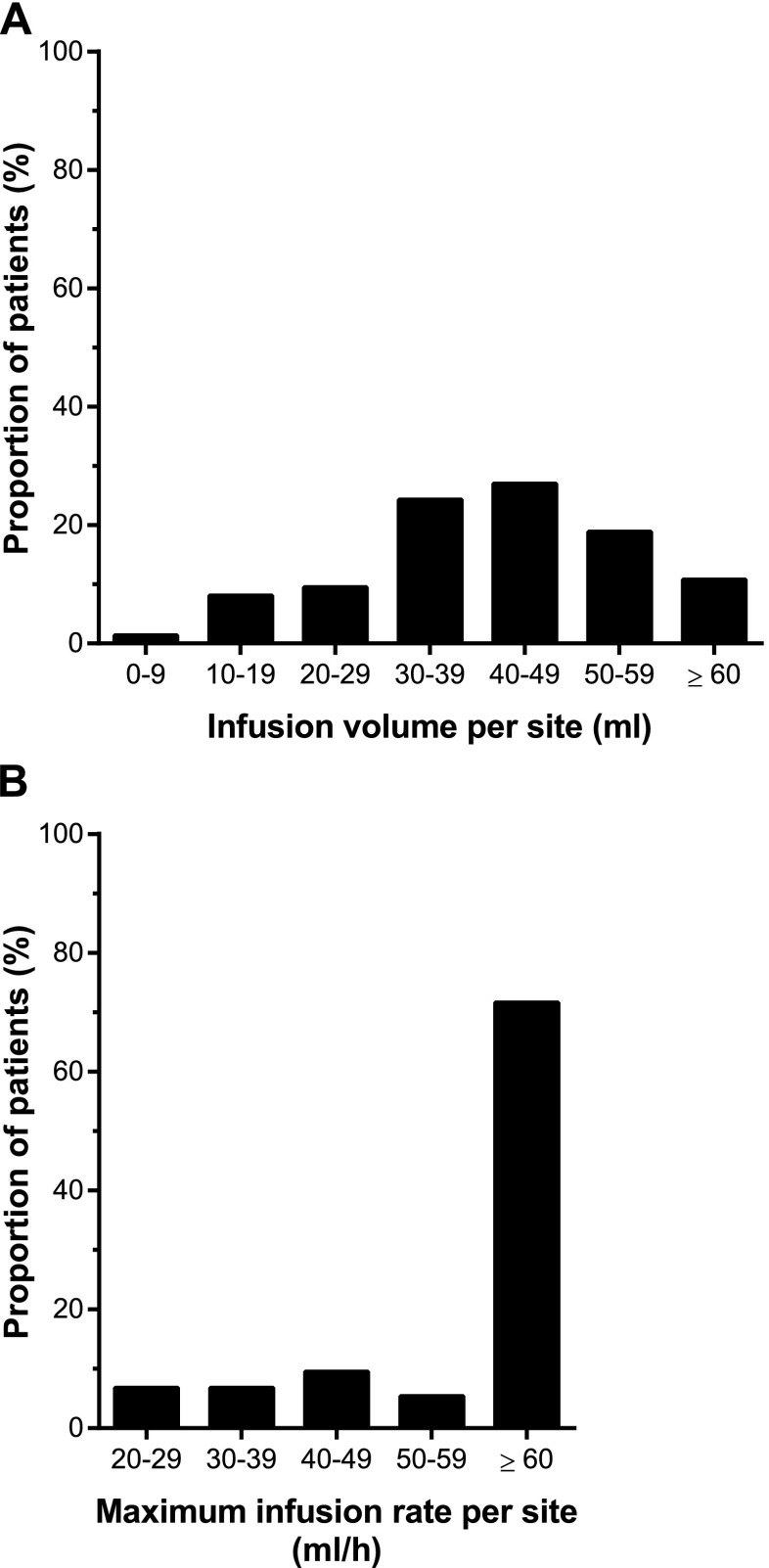
Categorization of patients by maximum infusion rate and infusion volume. **a** Infusion volume per site achieved at least once. **b** Maximum infusion rate achieved at least once

**Fig. 2:**
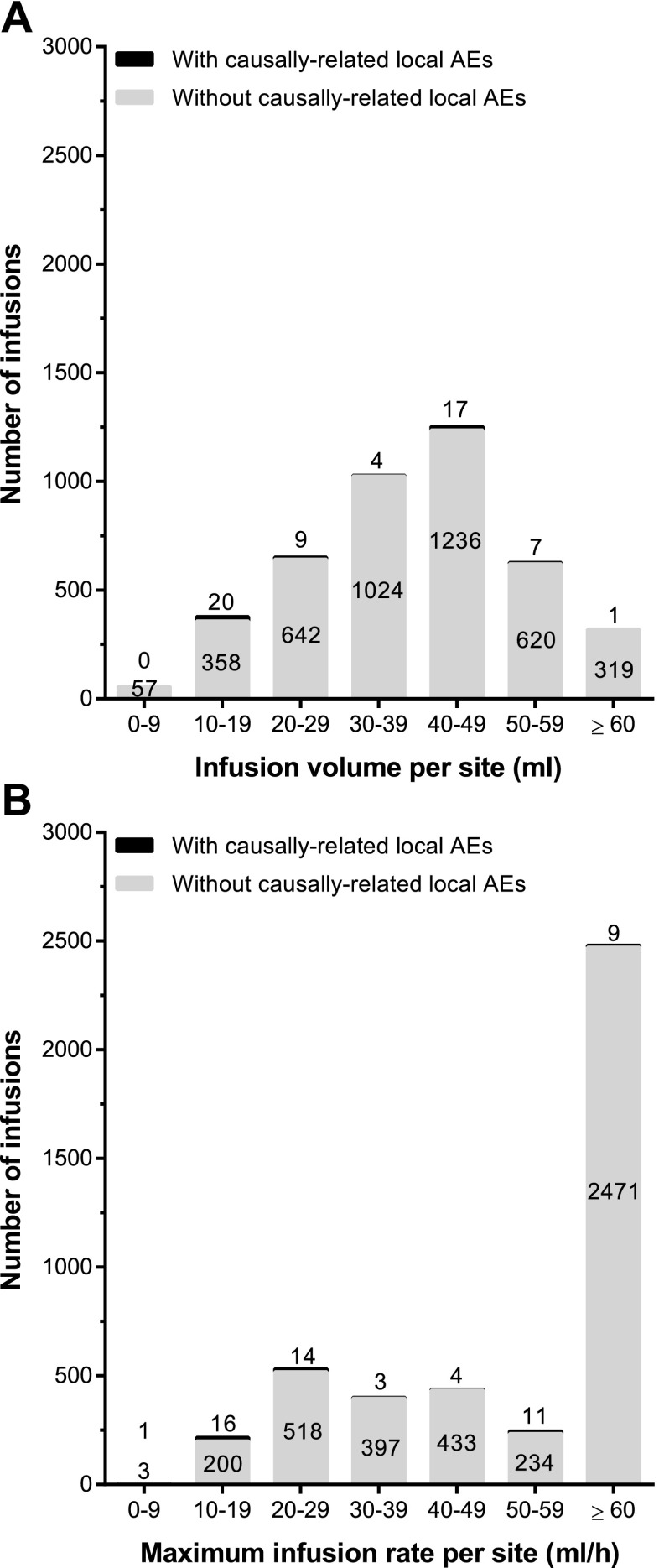
Tolerability of IGSC 20 % infusion rates and infusion volumes. **a** Infusion volumes. **b** Infusion rates. *Numbers above the bars* indicate the number of infusions associated with a causally related local AE and *numbers inside the bars* indicate the number of infusions not associated with any causally related local AE. Only infusions with complete infusion history (*n* = 4314) have been considered for these analyses

IGSC 20 % was administered to 74 patients for a median treatment duration of 380.5 days (range 30–629). At least one IGSC 20 % infusion was performed at home in 95.9 % of the patients, with or without professional assistance. Patients received a total of 4327 infusions of IGSC 20 % during the study, 79.1 % (3421/4327) of which were administered at home. Of note, certain visits had to be performed at the study site; therefore, even patients who had transitioned to home care were required per protocol to receive some infusions on site. The mean (±SD) weekly dose of IGSC 20 % was 0.222 ± 0.071 g/kg/week. Across all age groups, a median infusion volume per site of 39.50 ml (range 6.4–76.0) was administered (Table 4). For 74.8 % (3228/ 4314) of IGSC 20 % infusions, a volume ≥30 ml was infused per site. A volume of 60 ml and above per site was administered to 10.8 % of patients at least once (Fig. 1a) and in 7.4 % (320/4314) of IGSC 20 % infusions (Fig. 2a). Infusions with a volume per site < 60 ml usually had either a total required dose per infusion of <60 ml (one site) or a total volume of >60 ml and <120 ml and therefore were divided into two sites.

**Table 4:** Administration characteristics for IGSC 20 % by age group

Parameters^a^	Age group					All patients (*n* = 74)
	2 to <5 years (*n* = 1)	5 to <12 years (*n* = 14)	12 to <16 years (*n* = 8)	16 to <65 years (*n* = 45)	65 years and older (*n* = 9)	
Duration of infusions (h)						
Infusions (*n*)	50	718	346	2614	434	4162
Median	0.95	0.73	1.18	0.97	0.91	0.95
Min; max	(0.5; 1.4)	(0.3; 3.45)	(0.3; 3.5)	(0.2; 4.2)	(0.5; 6.4)	(0.2; 6.4)
Number of sites per infusion						
Infusions (*n*)	52	753	360	2700	461	4326
Median	1	2.0	2.0	2.0	2.0	2.0
Min; max	(1; 1)	(1; 3)	(1; 3)	(1; 4)	(1; 2)	(1; 4)
Maximum infusion rate per site (ml/h/ site)						
Infusions (*n*)	52	749	360	2692	461	4314
Median	15.0	30.0	50.0	60.0	60.0	60.0
Min; max	13.5; 20.0	4.4; 80.0	20.0;120.0	10.0; 180.0	5.0; 60.0	4.4; 180.0
Infusion volume per site (ml/site)						
Infusions (*n*)	52	749	360	2692	461	4314
Median	14.5	19.5	42.7	45.3	39.0	39.5
Min; max	13.5; 15.5	6.4; 43.0	19.2; 67.5	18.1; 76.0	31.8; 56.5	6.4; 76.0

^a^Only infusions with complete infusion parameters have been considered for each analyses

The median maximum rate of infusion was 60 ml/h/site (range 4.4–180) resulting in a median infusion duration of 0.95 h (range 0.2–6.4; Table 4). Overall, 71.6 % of patients achieved a maximum infusion rate of 60 ml/h/ site or more at least once (Fig. 1b) and 57.5 % (2480/4314) of infusions were administered at this infusion rate (Fig. 2b). A median number of 2.0 sites/infusion (range 1–4) were used for administration; 84.9 % (3662/4314) of infusions used two infusion sites or fewer.

### Tolerability

The short-term tolerability of IGSC 20 % treatment was evaluated by recording infusions for which the infusion rate had to be reduced, interrupted, or stopped due to tolerability concerns or AEs. For 99.8 % of IGSC 20 % infusions, there was no need to stop/interrupt administration or reduce the infusion rate (Table 5). The infusion rate had to be reduced in five (0.1 %) IGSC 20 % infusions administered to four patients, two of whom were children (aged 10 and 13 years, respectively). As a result of infusion leakage, two other pediatric patients (aged 8 and 11 years) each had one infusion interrupted, and one infusion was stopped in the 8-year old patient. IGSC 20 % infusions associated with a causally related local AE were categorized by volume and maximum infusion rate per site; the incidence of causally related local AEs did not increase at higher maximum infusion rates and infusion volumes (Fig. 2). Therefore, overall, a very strong positive tolerability profile for IGSC 20 % treatment was demonstrated with infusion rates and volumes of up to 60 ml/h/site and 60 ml per site, respectively.

**Table 5:** Infusions associated with tolerability concerns or AEs

	IGSC 20 % treatment dose			
	145 % of IGIV	Adjusted	Individualized	Overall
Total infusions (*n*)	731	867	2729	4327
Rate reduced, *n* (%)	1 (0.1)	4 (0.5)	0 (0.0)	5 (0.1)
Interrupted, *n* (%)	0 (0.0)	1 (0.1)	1 (0.0)	2(0.0)
Stopped, *n* (%)	0 (0.0)	0 (0.0)	1 (0.0)	1 (0.0)
No reduction, interruption or stop, *n* (%)	730 (99.9)	862 (99.4)	2727 (99.9)	4319 (99.8)

### Pharmacokinetic Parameters

The pharmacokinetics of serum IgG during IGSC 20 % treatment is depicted in Supplementary material Figure S4. During weekly IGSC 20 % administration at 145 % of the IGIV 10 % dose and at the individualized dose, no IgG peak was observed at day 1 postinfusion, and mean serum IgG levels remained constant throughout the treatment interval (Supplementary material Figure S4). Pharmacokinetic parameters determined for IGSC 20 % and IGIV 10 % are summarized in Table 6. The bioavailability of IGSC 20 % following 1.45 dose conversion and individual adjustment relative to IGIV 10 % was 1.09 (90 %CI 1.04 to 1.13, *n* = 49) as determined from the ratio of the geometric means of the AUC while on IGSC 20 % treatment once per week, compared to IGIV 10 % infusions (standardized to one week).

**Table 6:** Pharmacokinetic parameters for the IGSC 20 % and IGIV 10 % treatments

Treatment Period^a^ (IP)	Period 1 (IGIV 10 %)				Period 2 (IGSC 20 %, 145 % of IGIV 10 %)		Period 4 (IGSC 20 %, individualized)	
Dosing interval	3 weeks (*n* = 16)		4 weeks (*n* = 38)		1 week (*n* = 18)		1 week (*n* = 60)	
Parameter [unit]	Geom. mean	95 % CI	Geom. mean	95 % CI	Geom. mean	95 % CI	Geom. mean	95 % CI
AUC (g/days/l)	352.05	319.74–387.63	410.40	381.24–441.79	108.33	97.60–120.24	115.21	109.23–121.52
AUC / (Dose/Weight) [(g/days/l)/(g/kg)]	606.99	495.29–743.87	796.30	715.36–886.40	472.50	411.07–543.10	536.05	495.58–579.82
Clearance^b^ [ml/kg/days]	1.65	1.34–2.02	1.26	1.13–1.40	2.12	1.84–2.43	1.87	1.72–2.02
Cmax (g/l)	27.09	24.30–30.19	24.85	23.18–26.64	17.31	15.11–19.82	19.31	18.13–20.57
Tmax (h)	6.94	3.96–12.17	5.84	3.62–9.40	54.19	36.28–80.92	78.68	65.37–94.70
Cmin (g/l)	12.03	10.64–13.60	10.37	9.50–11.33	13.85	12.61–15.21	14.00	13.14–14.91

*95 %CI* 95 % confidence interval

^a^Period 1 and period 2 data includes patients aged 12 years and older; period 4 data includes patients aged 2 years and older.

^b^Apparent clearance for SC administration

### Total Serum IgG Trough Levels

Throughout IGSC 20 % treatment, median serum IgG trough values attained at the end of each treatment period remained above 14.5 g/L (Table 7). After 17 consecutive weeks of IGSC 20 % treatment at the individualized dose once per week, the median serum IgG trough levels were 15.23 g/L (95 %CI 13.59–15.70; *n* = 64). The median serum IgG trough levels recorded at the end of IGIV 10 % treatment administered every 3 weeks was 12.0 g/l (95 % CI 11.0–14.1 *n* = 19) and was 10.2 g/l (95 % CI 9.61–11.3; *n* = 50) at the end of IGIV 10 % treatment given every 4 weeks (Table 7).

**Table 7:** Trough levels of total IgG at the end of treatment periods

Treatment interval		Patient number (*n*)	Geometric mean (95 % CI)	Median (95 % CI)	Min; max
IGIV 10 %					
3 weeks		19	11.58 (10.36–12.94)	12.00 (11.00–14.10)	5.45;14.50
4 weeks		50	10.19 (9.55–10.88)	10.20 (9.61–11.30)	6.09;18.50
IGSC 20 %					
1 week	145 % of IV dose	27	14.77 (13.86–15.74)	15.30 (12.80–16.10)	12.00;20.30
1 week	Adjusted dose (145 % of IV dose)	66	14.20 (13.48–14.96)	14.65 (13.80–15.60)	8.65;22.20
1 week	Individualized dose^a^	57	14.74 (14.03–15.48)	15.10 (14.00–16.40)	8.81;23.40

*95 %CI* 95 % confidence interval

^a^Determined for each patient by comparing the individual serum IgG trough level attained in period 3 to the expected increase in serum IgG trough level calculated from the PK data from periods 1 and 2

### Patient Experience

Changes in treatment satisfaction were assessed for all patients when switching from IGIV 10 % in period 1 to IGSC 20 % in period 3 (adjusted dose) and at the end of period 4 (individualized dose).

Immunoglobulin-related treatment burden was evaluated with the LQI questionnaire in three domains: treatment interference, therapy-related problems, and therapy settings. No significant change in any of the domains was reported in patients aged 2–12 years. For patients 13 years and above, an improvement in each of the domains was observed between period 1 (IGIV 10 % administration) and the subsequent periods on IGSC 20 % treatment. Improvement in the treatment interference domain was found to be statistically significant (*p* = 0.008) across all age groups.

Assessment of treatment satisfaction using the TSQM-9 questionnaire found a significant improvement in the convenience domain (*p* < 0.001) between period 1 (IGIV 10 % administration) and period 4 (IGSC 20 % treatment). No significant improvement in the perception of effectiveness and global satisfaction was observed in either age group: 2–12 years and 13 years and older.

## Discussion

Subcutaneous (SC) administration of human polyclonal immunoglobulin preparations has been shown to be efficacious in preventing infection in patients with PIDD and is associated with fewer systemic adverse reactions compared to the IV route [5, 7, 23]. The improved systemic tolerability and sustained protection from infections observed are likely due to lower peak and higher trough levels of serum IgG administered SC [24]. Drawbacks, however, of SC compared to IV administration with the current standard of 5 and 10 % IgG preparations are that only small volumes can be infused and multiple infusion sites are required per week for treatment [5, 7]. A highly concentrated IgG preparation such as the present IGSC 20 % product may offer a new replacement therapy option for patients with PIDD as relatively large volumes per site could be infused at higher rates without impairing tolerability, thereby reducing infusion duration and number of infusion sites.

In this trial, a systemic exposure equivalent to the previous IGIV 10 % treatment was targeted; thus, the IGSC 20 % dose administered was adjusted to compensate for the lower bioavailability of IgG when administered SC [12]. SC administration of individually tailored IGSC 20% doses was efficacious in providing systemic exposure similar to that obtained with IV infusions. Serum IgG levels above 5 g/l are generally accepted as the minimal protective threshold although trough levels of at least 7 g/l may be required to achieve adequate protection against infections for some patients [25–27]. In the present study, trough levels were substantially higher than this accepted protective threshold with a median serum IgG trough level above 14 g/l throughout IGSC 20 % treatment. This may have been due in part to the relatively high Ig doses that patients had been receiving prior to study onset. Indeed, as per study design, the patients received the same monthly IgG dose/kg in period 1 as they did prior to study entry. Therefore, the dose of IGIV 10 % was not predefined per protocol but was determined by the treating physician and the IGSC 20 % dose was subsequently adjusted to achieve systemic exposure equivalent to the IGIV 10 % treatment dose. Although high, the mean weekly dose of IGSC 20 % dose in this study was within the range (0.177–0.224 g/kg BW per week) reported for another IGSC 20 % product [11].

The high serum IgG trough levels maintained throughout the study were efficacious in preventing VASBIs as demonstrated by the low annualized rate of VASBIs per patient during IGSC 20 % treatment. This rate of VASBIs was significantly lower than the threshold specified by the FDA and EMA guidelines as providing substantial evidence of efficacy [19, 28, 29]. A further indicator of the protective effect of IGSC 20 % was the annualized frequency of any infections (2.41 events/patient), which was comparable to the incidence of infections (2.76 events /patient) reported for another IGSC 20 % preparation [11] and lower than the annualized rate of any infection episodes reported with other licensed IGSC products: 3.946 events/patient in a 6-month study with IGSC 16 % [30] or 4.1 events/patient with an IGSC 10 % product [12], although differences in study design and product concentrations may limit direct comparison. These efficacy results, together with the positive outcomes obtained for the additional efficacy assessments (number and duration of fever episodes and of hospitalizations as well as the low rate of days missed from work, school, or daily activity), further support the protective effect of IGSC 20 % as replacement therapy in PIDD.

While the SC administration of IgG has been associated with fewer systemic AEs than IGIV, a higher incidence of local AEs has been reported [31]. The incidence of systemic AEs related to IGSC 20 % infusions in the present study was about 11-fold lower than for IGIV 10 % treatment. The rate per infusion of local AEs deemed related to IGSC 20 % (0.015 event/infusion) was much lower than the rates reported with a licensed equivalent IGSC 20 % preparation in studies conducted in the USA (0.592 and 0.600 event/infusion, respectively) [11, 32] and in Japan (0.274 event/infusion) [33] and lower than the rates observed in an EU study (0.060 event/infusion) [34]. Consistent with these data, IGSC 20 % treatment was well tolerated, with mostly mild (92.5 %) or moderate (7.5 %), and no severe related local AEs reported. No patients discontinued due to a local adverse reaction, and 98.7 % of IGSC 20 % infusions were not associated with any local related non-serious AE. For 99.8 % of IGSC 20 % infusions, no reduction of infusion rate was required; no infusions had to be interrupted or stopped due to AE or tolerability concerns, indicating an overall short-term tolerability at least equivalent to that observed with a similar licensed IGSC 20 % product [33]. The same model of electromechanical syringe-driver pump was used in all patients to exclude potential differences in tolerability and local adverse reactions that could arise from different pump selections. Electromechanical syringe-driver pumps were also used in US and EU studies conducted with a licensed IGSC 20 % product [11, 34].

The favorable tolerability profile of IGSC 20 % infusions permitted the administration of higher infusion rates and volumes per site: the median maximum IGSC 20 % infusion rate (60 ml/h/site; range 4.4–180) was above the maximum infusion rate recommended for another IGSC 20 % preparation (15–25 ml/h/site) [35]. This infusion rate was achieved in more than half of completed IGSC 20 % infusions without increase in the proportion of infusions associated with local adverse reactions, indicating that the maximum recommended infusion rate of 60 ml/h/site for this study was well-tolerated. Of note, there was no predominance of any body mass index in patients across all age groups; therefore, the possibility that the favorable tolerability of higher rates and volumes was related to the amount of subcutaneous adipose tissue is unlikely. The similarity of IGSC 20 % osmolality (280–292 mOsm/kg) with the physiological plasma osmolality (280–296 mOsm/kg) could partly account for the positive tolerability profile of IGSC 20 % [36, 37]. By comparison, the osmolality of a licensed IGSC 20 % product is higher (380 mOsm/kg) [38]. The use of glycine rather than proline as stabilizer in the investigated IGSC 20 % product may also contribute to its improved tolerability.

The high infusion rates enabled a lower infusion duration (median 0.95 h, range 0.2–6.4 h) for IGSC 20 %, which is markedly shorter than the duration reported for weekly infusions of a licensed IGSC 20 % preparation (2 h; range 0.5–17.0 h) [11]. In addition, infusion volumes of up to 60 ml/site were administered in this study, resulting in one or two sites per IGSC 20 % administration in over 75 % of infusions compared to five or fewer sites in 75 % of infusions with a licensed IGSC 10 % product [12]. The viscosity of IGSC 20 % is similar to those of licensed IGSC 16 and 20 % preparations (14.4 versus 14.4 mPa/s and 14.7 mPa/s, respectively). In addition, there was no specified needle length for infusions. Regardless, infusion characteristics determined for the study product favored shorter infusion duration and fewer infusion sites. Infusion sites were rotated to avoid any single infusion site being used repeatedly within a short time interval.

Patients across all ages adhered to the SC administration of treatment as evidenced by the overall high rate of study completion; 90 % of patients treated with IGSC 20 % completed the study, including 95 % of patients aged 2 to <16 years old, suggesting that IGSC 20 % did not place an unreasonable burden on the daily activities of the adult and pediatric patients. Results of patient-reported outcome measures also showed an overall positive evaluation of IGSC 20 % treatment; in particular, a significant increase in treatment satisfaction in terms of treatment convenience and interference was observed. Home infusion was adopted by a high proportion (96 %) of patients and may have contributed to the perception of enhanced convenience. These results, in line with several reports from other studies, show that the treatment experience of patients with PIDD improves as a result of the practice of subcutaneous administration (reviewed by Wasserman [5]).

In conclusion, IGSC 20 % administered SC at an individually adjusted dose was shown to have an excellent safety and tolerability profile in patients with PIDD. In addition, the low incidence of infections and the maintenance of protective trough levels for total serum IgG demonstrate the efficacy of IGSC 20 % treatment. Excellent tolerability across all age groups enabled infusions to be administered at higher rates and volumes compared to conventional SC preparations, leading to shorter infusion durations and fewer infusion sites with a reflection onto patient experience in terms of treatment interference and convenience.

## Electronic supplementary material

Below is the link to the electronic supplementary material.

ESM 1 (DOCX 246 kb)
