# Supplementary material for: Efficacy, Safety, and Pharmacokinetics of a Novel Human Immune Globulin Subcutaneous, 20 % in Patients with Primary Immunodeficiency Diseases in North America
Source: J Clin Immunol. 2016 Aug 31;36(7):700–12. doi: 10.1007/s10875-016-0327-9 (PMC5018260; doi:10.1007/s10875-016-0327-9)
Supplement: Supplementary file 1 — (DOCX 246 kb) [file 10875_2016_327_MOESM1_ESM.docx]

# SUPPLEMENTARY MATERIAL

**TITLE:** Efficacy, safety and pharmacokinetics of a novel human immune globulin subcutaneous, 20% in patients with primary immunodeficiency diseases in North America

**Authors:**

Daniel Suez^1^, Mark Stein^2^, Sudhir Gupta^3^, Iftikhar Hussain^4^, Isaac Melamed^5^, Kenneth Paris^6^, Amy Darter^7^, Christelle Bourgeois,^8^ Sandor Fritsch,^8^ Heinz Leibl,^8^ Barbara McCoy,^9^ David Gelmont^10^ and Leman Yel^9, 3,*^

**Affiliations**

^1^ Allergy, Asthma & Immunology Clinic PA, Irving TX, USA

^2^ Allergy Associates of the Palm Beaches, North Palm Beach FL, USA

^3^ Division of Basic and Clinical Immunology, University of California at Irvine, Irvine CA, USA

^4^ Vital Prospects Clinical Research Institute, Tulsa OK, USA

^5^ IMMUNOe International Research Centers, Centennial CO, USA

^6^ LSU Health Sciences Center, Children’s Hospital, New Orleans LA, USA

^7^ Oklahoma Institute of Allergy & Asthma Clinical Research, LLC, Oklahoma City OK, USA

^8^ Baxalta Innovations GmbH, now part of Shire, Vienna, Austria

^9^ Baxalta US Inc., now part of Shire, 650 East Kendall Street, Cambridge MA, USA

^10^ Baxalta US Inc., now part of Shire, Westlake Village, CA, USA

***Corresponding Author**

Leman Yel, MD

Sr. Medical Director, Global Clinical Science Immunology, Research and Development

Baxalta US Inc., now part of Shire
650 East Kendall Street
Cambridge, MA 02142 USA

Tel: +1 (617) 588-8437

Email: Leman.Yel@shire.com

ELIGIBILITY CRITERIA

1. **Inclusion criteria**

Patients who met **ALL** of the following criteria were eligible for this study:

1. Patient had a documented diagnosis of a form of primary humoral immunodeficiency involving defective antibody formation and requiring gammaglobulin replacement, as defined according to the IUIS Scientific Committee, 2011 [[1]](#_References) and by diagnostic criteria according to Conley et al. (1999) [[2]](#_References). The diagnosis had to be confirmed by the Medical Director prior to first treatment with IP in the study.
2. Patient was 2 years or older at the time of screening, and had a minimum body weight of 13 kg.
3. Written informed consent was obtained from either the patient or the patient’s legally authorized representative prior to any study-related procedures and study product administration.
4. Patient had received a stable monthly equivalent dose (i.e. without need for dose adjustment due to lack of efficacy or low trough IgG levels) of IgG at an average minimum dose equivalent to 300 mg/kg BW/4 weeks and a maximum dose equivalent to 1.0 gram/kg BW/4 weeks for a minimum of 12 weeks prior to first treatment with IP in the study. The reason for using doses > 600mg/kg BW/4 weeks was to be documented on the appropriate CRF. Examples of pre-study dosing frequency:
5. IV at mean intervals of approximately 3 or 4 weeks or
6. SC at mean intervals of approximately 1 or 2 weeks
7. SC alternative treatment schedule (e.g. 2x/week)
8. Patient had a serum trough level of IgG > 500 mg/dL at screening.
9. Patient was willing and able to comply with the requirements of the protocol
10. **Exclusion Criteria**

Patients who met **ANY** of the following criteria were not eligible for this study:

1. Patient had a known history of or was positive at screening for one or more of the following: hepatitis B surface antigen (HBsAg), polymerase chain reaction (PCR) for hepatitis C virus (HCV), PCR for human immunodeficiency virus (HIV) Type 1/2.
2. Abnormal laboratory values at screening that met any one of the following criteria (abnormal tests could be repeated once to determine if they persisted):
3. Persistent alanine aminotransferase (ALT) and aspartate amino transferase (AST) > 2.5 times the upper limit of normal for the testing laboratory
4. persistent severe neutropenia (defined as an absolute neutrophil count [ANC] ≤ 500 /mm^3^)
5. Patient had creatinine clearance (CLcr) value that was < 60% of normal for age and gender, either measured, or calculated according to the Cockcroft-Gault [3] formula^^[[1]](#footnote-1)^^:
6. Patient had been diagnosed with or had a malignancy (other than adequately treated basal cell or squamous cell carcinoma of the skin or carcinoma in situ of the cervix), unless the disease-free period prior to screening exceeded 5 years.
7. Patient was receiving anti-coagulation therapy (low dose aspirin, ≤ 325 mg/day, is permitted) or had a history of thrombotic episodes (including deep vein thrombosis, myocardial infarction, cerebrovascular accident, pulmonary embolism) or sickle cell disease with crisis within 12 months prior to screening or had a history of thrombophilia.
8. Patient had abnormal protein loss (protein losing enteropathy, nephrotic syndrome).
9. Patient had anemia that would preclude phlebotomy for laboratory studies according to standard practice at the site.
10. Patient had had an acute serious bacterial infection within the 3 months prior to screening as defined in the FDA Guidance for Industry (June 2008) [4].
11. Patient had an ongoing history of hypersensitivity or persistent reactions (urticaria, breathing difficulty, severe hypotension, or anaphylaxis) following IV immunoglobulin, SC immunoglobulin, and/or Immune Serum Globulin (ISG) infusions.
12. Patient had severe immunoglobulin A (IgA) deficiency (less than 0.07g/L) with known anti-IgA antibodies and a history of hypersensitivity.
13. Patient was on continuous systemic antibacterial antibiotics at doses sufficient to treat or prevent bacterial infections, and, in the opinion of the PI, could not stop these for the duration of the study without putting the patient at risk of increased infections.
14. Patient had active infection and was receiving antibiotic therapy for the treatment of infection at the time of screening.
15. Patient had a bleeding disorder or thrombocytopenia with a platelet count less than 20,000/µL, or who, in the opinion of the investigator, was at significant risk of increased bleeding or bruising as a result of SC therapy.
16. Patient had total protein > 9 g/dL or myeloma or macroglobulinemia (IgM) or paraproteinemia.
17. Patient had severe dermatitis that would preclude adequate sites for safe product administration.
18. Women of childbearing potential meeting any one of the following criteria
19. patient presented with a positive pregnancy test
20. patient was breast feeding
21. patient intended to begin nursing during the course of the study
22. patient did not agree to employ adequate birth-control measures (e.g. intrauterine device, diaphragm or condom [for male partner] with spermicidal jelly or foam, or birth control pills/patches) throughout the course of the study
23. Patient had participated in another clinical study and had been exposed to an investigational product (IP) or device within 30 days prior to study enrollment (exception: treatment in a previous Baxalta, now part of Shire immunoglobulin study).
24. Patient was scheduled to participate in another (non-Baxalta, now part of Shire) non-observational (interventional) clinical study involving an IP or device during the course of the study.

SUPPLEMENTARY TABLES

**Table S1: Demographic and baseline characteristics of treated patients**

| **Parameter** | **Category/ *Statistics*** | **Patients per age group** | | | | | **Total N = 77 n (%)** | |
| --- | --- | --- | --- | --- | --- | --- | --- | --- |
|  |  | **2 to <5 years^a^ N = 1 n (%)** | **5 to <12 years^a^ N = 14 n (%)** | **12 to <16 years^a^ N = 8 n (%)** | **16 to <65 years^a^ N = 45 n (%)** | **65 years and older^a^ N = 9 n (%)** |  |  |
| **Gender** | Male | 1 (100.0) | 13 (92.9) | 7 (87.5) | 18 (40.0) | 1 (11.1) | 40 (51.9) | |
|  | Female | 0 (0.0) | 1 (7.1) | 1 (12.5) | 27 (60.0) | 8 (88.9) | 37 (48.1) | |
| **Age (years)** | Median  Min; max | 3.0  3; 3 | 8.0  6; 11 | 13.0  12; 15 | 48.0  16;63 | 69.0  66; 83 | 36.0  3; 83 | |
| **Weight (kg)** | Median  Min; max | 13.20  13.2; 13.2 | 25.85  19.6; 56.1 | 50.50  39.9; 80.0 | 76.00  42.2; 161.8 | 68.20  48.0; 113.4 | 68.20  13.2; 161.8 | |
| ^a^Age at screening. | | | | | | | | |

**Table S2: Primary immunodeficiency diagnoses**

| **System Category^a^** | | **n of N (%)** | |
| --- | --- | --- | --- |
| Congenital agammaglobulinemia - XLA | | 9 of 77 (11.7) | |
| Agammaglobulinemia – autosomal recessive | | 2 of 77 (2.6) | |
| X-linked hyper IgM (XHIM) | | 1 of 77 (1.3) | |
| Hyper-IgM - autosomal recessive | | 1 of 77 (1.3) | |
| Severe combined immune deficiency | | 1 of 77 (1.3) | |
| Common variable immune deficiency^b^ | | 26 of 77 (33.8) | |
| Specific antibody deficiency^c^ | | 18 of 77 (23.4) | |
| Specific antibody deficiency with IgG subclass deficiency^c^ | | 7 of 77 (9.1) | |
| Specific antibody deficiency with hypogammaglobulinemia | | 10 of 77 (13.0) | |
| Ataxia telangiectasia | | 1 of 77 (1.3) | |
| Other: IgG1 and IgG3 subclass deficiency with low IgG | | 1 of 77 (1.3) | |
| Total | | 77 of 77 (100.0) | |
| ^a^ The diagnosis of PIDD involving defective antibody production and requiring IgG replacement as defined by the IUIS Scientific Committee (2011) [1]was performed using diagnostic criteria according to Conley et al. (1999) [2].  ^b^ One patient diagnosed with CVID had a familial mutation of the gene TNFRSF13B encoding the transmembrane activator and calcium-modulator and cyclophilin-ligand interactor (TACI) a member of the tumor necrosis factor-receptor superfamily. For all other patients the genetic defects were unknown.  ^c^ All patients diagnosed with specific antibody deficiency with hypogammaglobulinemia had low serum IgG levels and normal serum IgA and IgM levels. | |  |  |

SUPPLEMENTARY FIGURES

**Figure S1: Study design**

**Study Enrollment**

**Period 1**

IGIV 10%
13 weeks
PK assessment at 2^nd^ to last infusion

**Period 2**

IGSC 20%

145% of IV dose

Up to 12-16 weeks (until „adjusted dose“ was available)

PK assessment at 9^th^ infusion

Determine „adjusted dose“ for Period 3

**End of Study**

**Period 4**

IGSC 20%

Individualized dose

40 weeks

PK Assessment at Infusion 17

**Period 3^a^**

IGSC 20%

Adjusted dose

12 weeks

Assessment of steady-state serum IgG level at Infusion 9

Determine „individualized dose“ for Period 4

**Figure S2: Patient disposition**

Discontinued from Period 4^d^
N = 1

Discontinued from Period 1^a^
N = 2

Discontinued from Period 4^e^
N = 2

**2 - 15 years**N = 23

**>15 years**N = 54

**Started Period 1
IGIV 10%**

N = 77

**2 - 15 years**N = 4

**>15 years**N = 47

**>15 years**N = 41

**Continued in Period 3**N = 29

N = 77

**>15 years**N = 53

**Started Period 3**

**IGSC 20 %**

N = 74

**2 - 15 years**N = 21

**2 - 15 years**N = 21

**Started Period 4**

**IGSC 20 %**

N = 70

**>15 years**N = 49

**Completed Period 4
IGSC 20 %**

N = 67

Discontinued from Period 1^b^
N = 1

**Started Period 2
IGSC 20 %**

N = 45

Discontinued from Period 3^c^
N = 4

**2 - 15 years**N = 20

**Figure S3: Related local AEs reported over time during IGSC 20% treatment**

**
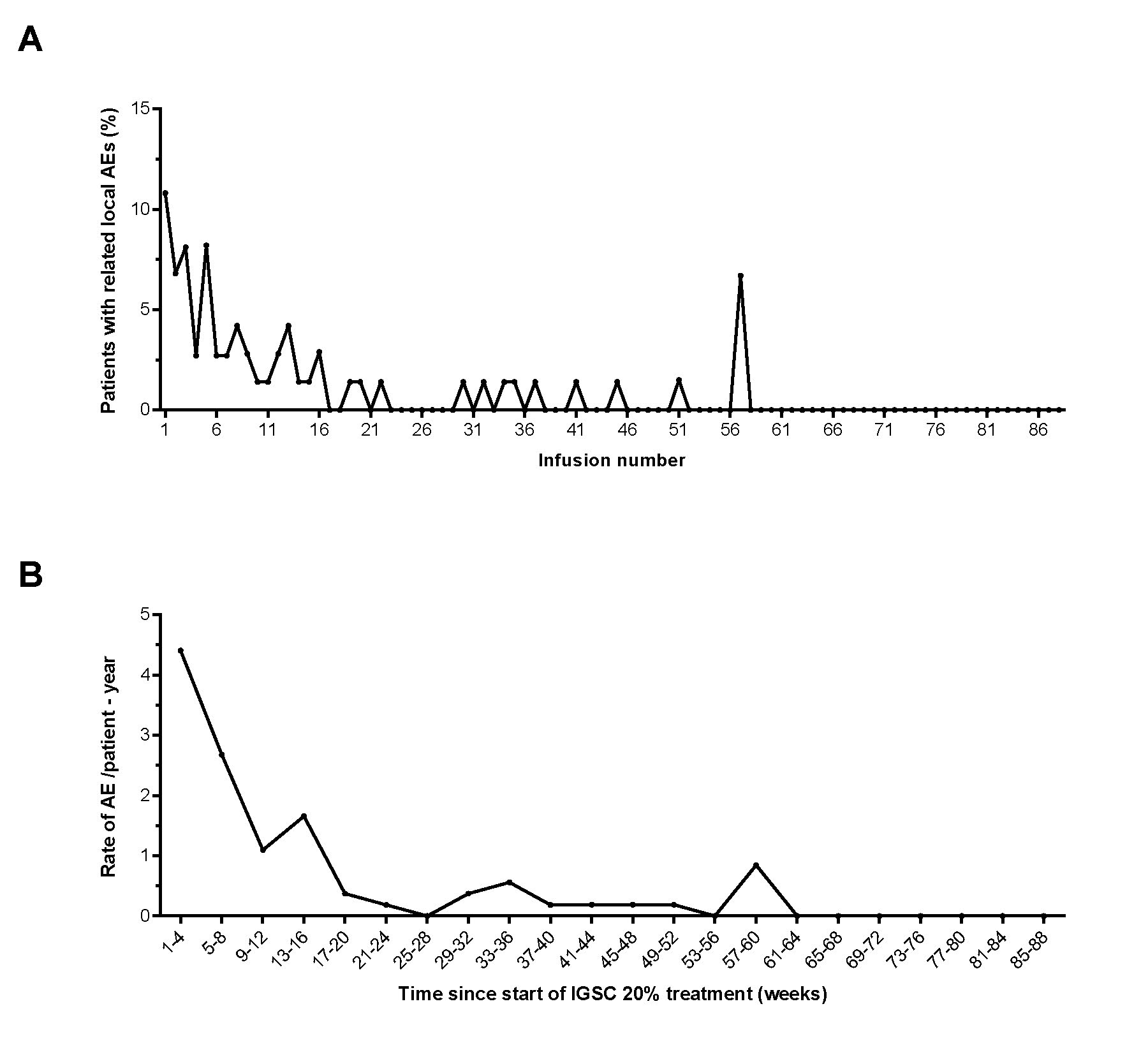
**

**Figure S4: Pharmacokinetic of IgG levels over the course of a treatment interval**


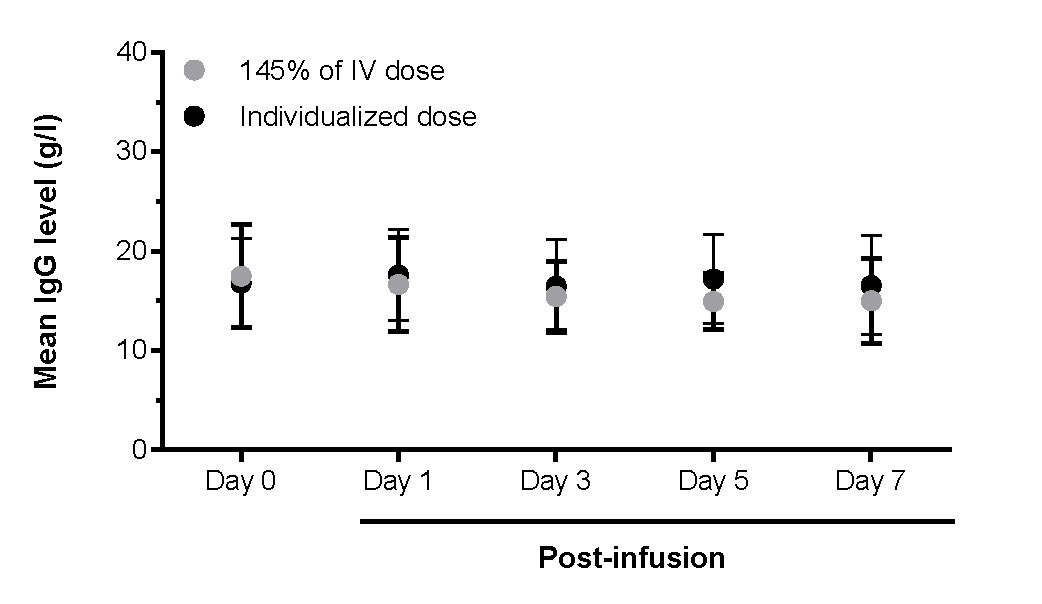


**SUPPLEMENTARY FIGURE CAPTIONS**

**Figure S1: Study design**

**^a^** Treatment in Period 3 started as soon as the “adjusted dose” became available. Consequently, patients enrolled later after the Adjusted Dose had become available went directly into treatment with the Adjusted Dose (Period 3).

**Figure S2: Patient disposition**

^a^ One patient was discontinued due to non-adherence and one patient because of an AE related to IGIV 10% administration

^b^ Patient was discontinued due to non-adherence.

^c^ One patient experienced an AE assessed as unrelated to IGSC 20% treatment and withdrew consent and 3 patients withdrew consent for personal reasons

^d^ Patient withdrew consent for personal reasons

^e^ One patient was discontinued due to non-adherence and one patient withdrew consent for personal reasons

**Figure S3: Related local AEs reported over time during IGSC 20% treatment**

**A)** Proportion of patients reporting related local AEs at each infusion; **B)** Annualized rate of related local AEs over time.

Planned treatment period was variable according to enrollment time point, with a minimum of 52 weeks.

**Figure S4: Pharmacokinetic of IgG levels over the course of a treatment interval**

Samples were collected on Day 0 within 60 min prior to the first IGSC 20% infusion and on Days 1, 3, 5 and 7 post-infusion (± 6 hours from infusion start). Mean serum IgG concentrations in patients treated with IGSC 20% at the 145% of the IGIV 10% dose and at the individualized dose are plotted. Vertical bars represent standard deviations.

REFERENCES

1. Al-Herz W, Bousfiha A, Casanova JL, Chapel H, Conley ME, Cunningham-Rundles C and others. Primary immunodeficiency diseases: an update on the classification from the International Union of Immunological Societies Expert Committee for Primary Immunodeficiency. Front Immunol. 2011; 2:54.

2. Conley ME, Notarangelo LD, Etzioni A. Diagnostic criteria for primary immunodeficiencies. Representing PAGID (Pan-American Group for Immunodeficiency) and ESID (European Society for Immunodeficiencies). Clin Immunol. 1999; 93:190-197.

3. Cockcroft DW, Gault MH. Prediction of creatinine clearance from serum creatinine. Nephron. 1976; 16:31-41.

4. U.S.Department of Health and Human Services, Food and Drug Administration, and Center for Biologics Evaluation and Research. Guidance for industry: Safety, efficacy, and pharmacokinetic studies to support marketing of immune globulin intravenous (human) as replacement therapy for primary humoral immunodeficiency. 17. 2008. U.S. Department of Health and Human Services, Food and Drug Administration (FDA).
Ref Type: Report

1. (140 – Age [in years]) x Body weight (in kg)
    for males: CL_cr_ = ---------------------------------------------------------------------
    72 x Serum creatinine (in mg / dL)

    (140 – Age [in years]) x Body weight (in kg) x 0.85
    for females: CL_cr_ = ---------------------------------------------------------------------
    72 x Serum creatinine (in mg / dL) [↑](#footnote-ref-1)
